# Continental Island Formation and the Archaeology of Defaunation on Zanzibar, Eastern Africa

**DOI:** 10.1371/journal.pone.0149565

**Published:** 2016-02-22

**Authors:** Mary E. Prendergast, Hélène Rouby, Paramita Punnwong, Robert Marchant, Alison Crowther, Nikos Kourampas, Ceri Shipton, Martin Walsh, Kurt Lambeck, Nicole L. Boivin

**Affiliations:** 1 Department of Sociology & Anthropology, Saint Louis University, Madrid, Spain; 2 Laboratoire de Géologie de l’École Normale Supérieure, UMR 8538 du CNRS, 75231 Paris, France; 3 Research School of Earth Sciences, The Australian National University, Canberra, ACT 0200, Australia; 4 Faculty of Environment and Resource Studies, Mahidol University, Salaya, Nakhon Pathom 73170, Thailand; 5 Environment Department, York Institute for Tropical Ecosystems, York, United Kingdom; 6 School of Social Science, The University of Queensland, Brisbane, Australia; 7 Office of Lifelong Learning, University of Edinburgh, Edinburgh, Scotland, United Kingdom; 8 Biological and Environmental Sciences, University of Stirling, Stirling, Scotland, United Kingdom; 9 McDonald Institute for Archaeological Research, University of Cambridge, Cambridge, United Kingdom; 10 British Institute in Eastern Africa, British Academy, Nairobi, Kenya; 11 Wolfson College, University of Cambridge, Cambridge, United Kingdom; 12 Research Laboratory for Archaeology and the History of Art, School of Archaeology, University of Oxford, Oxford, United Kingdom; 13 Max Planck Institute for the Science of Human History, Jena, Germany; Universidade do Algarve, PORTUGAL

## Abstract

With rising sea levels at the end of the Pleistocene, land-bridge or continental islands were formed around the world. Many of these islands have been extensively studied from a biogeographical perspective, particularly in terms of impacts of island creation on terrestrial vertebrates. However, a majority of studies rely on contemporary faunal distributions rather than fossil data. Here, we present archaeological findings from the island of Zanzibar (also known as Unguja) off the eastern African coast, to provide a temporal perspective on island biogeography. The site of Kuumbi Cave, excavated by multiple teams since 2005, has revealed the longest cultural and faunal record for any eastern African island. This record extends to the Late Pleistocene, when Zanzibar was part of the mainland, and attests to the extirpation of large mainland mammals in the millennia after the island became separated. We draw on modeling and sedimentary data to examine the process by which Zanzibar was most recently separated from the mainland, providing the first systematic insights into the nature and chronology of this process. We subsequently investigate the cultural and faunal record from Kuumbi Cave, which provides at least five key temporal windows into human activities and faunal presence: two at the end of the Last Glacial Maximum (LGM), one during the period of post-LGM rapid sea level rise and island formation, and two in the late Holocene (Middle Iron Age and Late Iron Age). This record demonstrates the presence of large mammals during the period of island formation, and their severe reduction or disappearance in the Kuumbi Cave sequence by the late Holocene. While various limitations, including discontinuity in the sequence, problematize attempts to clearly attribute defaunation to anthropogenic or island biogeographic processes, Kuumbi Cave offers an unprecedented opportunity to examine post-Pleistocene island formation and its long-term consequences for human and animal communities.

## Introduction

Rising seas at end of the Last Glacial Maximum (LGM) in the terminal Pleistocene transformed coastlines, drowning land bridges and leaving islands in their wake. Particularly notable examples of continental or land-bridge islands are found in modern-day Indonesia, New Guinea, Tasmania, and Britain, but numerous smaller islands were also formed along coastlines across the globe. The isolation of nascent islands can have significant impacts on fauna [1–4], including loss of species diversity through a process known as faunal relaxation. Most studies of faunal relaxation, however, come from modern cases, often artificial “islands” formed through damming or habitat fragmentation, for example [5–9]. Fossil records for recently formed continental islands are rare [10–12], and in many cases, studies of faunal change are complicated by human colonization [13–15]. Long-term paleoecological and archaeological records are therefore critical to address the complex interaction of influences on island biota, including island biogeography, climate change, and anthropogenic habitat modification, hunting, and species translocations. An understanding of the long-term dynamics of faunal change is not only essential to address these issues, but is also central to the conservation of island biodiversity today [15], particularly in an era of intensive human-mediated defaunation [16].

In the western Indian Ocean, studies of island biogeography and the impacts of human settlement on faunal biota have been largely limited to the late Holocene and have focused on Madagascar [17–19] or oceanic islands such as the Mascarenes [20–24] and Comoros [21, 25–28]. The continental islands of eastern Africa, including the Lamu, Mafia, and Zanzibar archipelagos, lack faunal records with sufficient time-depth to permit analysis of the effects of post-LGM island formation, with one important exception. Archaeological fieldwork at Kuumbi Cave (S 6°21’40”, E 39°32’33”), on Zanzibar Island (also known as Unguja Island), has revealed the deepest cultural and faunal sequence for any island in the region. This site offers a unique temporal perspective, enabling an examination of faunal change and human impacts during the terminal Pleistocene and Holocene.

Zanzibar is the largest island in the eponymous archipelago. Unlike the second-largest island of Pemba, which has been isolated since at least the early Pliocene [29] (cf. [30]), Zanzibar has been periodically part of the mainland during sea level lowstands associated with Quaternary glaciations. Zanzibar would, however, have been an island during most Quaternary interglacial periods and was most recently separated from the mainland when sea levels rose following the LGM. Zanzibar is also by far the best-documented island in the region in terms of geology, contemporary flora and fauna, and ethnography of recent hunting and animal consumption practices [28, 31–35]. This is largely thanks to Zanzibar’s longstanding prominence in global economies, illustrated by its role in, for example, the premodern spice trade and, more recently, global tourism [36–37]. Zanzibar thus makes an excellent case study for exploring the effects of island formation and human occupation on animal populations.

Despite this research history, there are major gaps in our knowledge of Zanzibar. The first is the temporal and spatial sequence of the island’s separation from the mainland following the LGM, and the establishment of the economically important mangroves and reefs that fringe the island. While the separation of Zanzibar has been discussed in general terms [29, 34, 38], no detailed study of its terminal Pleistocene-early Holocene formation has been made, even though this is imperative to understanding the island’s history and biogeography, as well as its human occupations. The environmental history of the island is also underexplored, though a recent series of studies of Holocene sea-level changes has begun to reveal their possible effects on Zanzibar’s coastal habitats [39–42]. Finally, due to a paucity of systematic paleontological and zooarchaeological studies, little is known of Zanzibar’s long-term faunal history, the exception being a report limited to fossil invertebrates [43] (cf. [32]). A multidisciplinary approach, drawing on earth science, environmental science and archaeology, can address these lacunae to begin to elucidate the interplay of natural and anthropogenic factors that have shaped Zanzibar’s ecosystems.

In this paper, we present a reconstruction of the sea level changes that created Zanzibar Island after the LGM, informed by field and modeling studies. We examine these in relation to the findings of a zooarchaeological analysis of faunal remains from the 2012 excavations at Kuumbi Cave [44–46]. This is the earliest published archaeological site anywhere on the eastern African coast south of the Horn, and provides at least five key temporal windows into human activities and faunal presence: two during the Late Pleistocene, when Zanzibar was part of the mainland; one during the period of post-LGM rapid sea level rise and island formation; and two in the late Holocene, when the arrival of farmers and herders from the mainland brought new changes to the island. Kuumbi Cave was previously explored through several campaigns [47–49], but the 2012 excavations revised and redated the stratigraphic sequence, and placed emphasis on the recovery of bioarchaeological material, especially plant and animal remains [44]. In these most recent excavations, Kuumbi Cave was dated using high-precision radiocarbon and luminescence methods applied to 20 samples and interpreted in light of sediment deposition processes.

Our investigation faces some important limitations, in particular due to discontinuity across the Kuumbi Cave sequence, resulting in discrete temporal windows rather than a continual, long-term cultural and faunal record. The faunal assemblage also represents a limited sample from only one site, whereas a broader sample across multiple sites would be ideal to draw conclusions about island-wide processes. On the other hand, uninterrupted cave sequences are exceedingly rare, and Kuumbi Cave’s long sequence has not so far been replicated at other island sites in the region. In addition, the site’s faunal assemblage is unprecedented for this region, both in terms of its antiquity and the chronometric and zooarchaeological analyses to which it has been subjected. Bearing in mind the above caveats, this assemblage offers an invaluable resource for looking at island biogeography from a deeper temporal perspective, and exploring the interplay between past processes of island formation, human activity, and defaunation.

## The Research Setting

**Fig 1:**
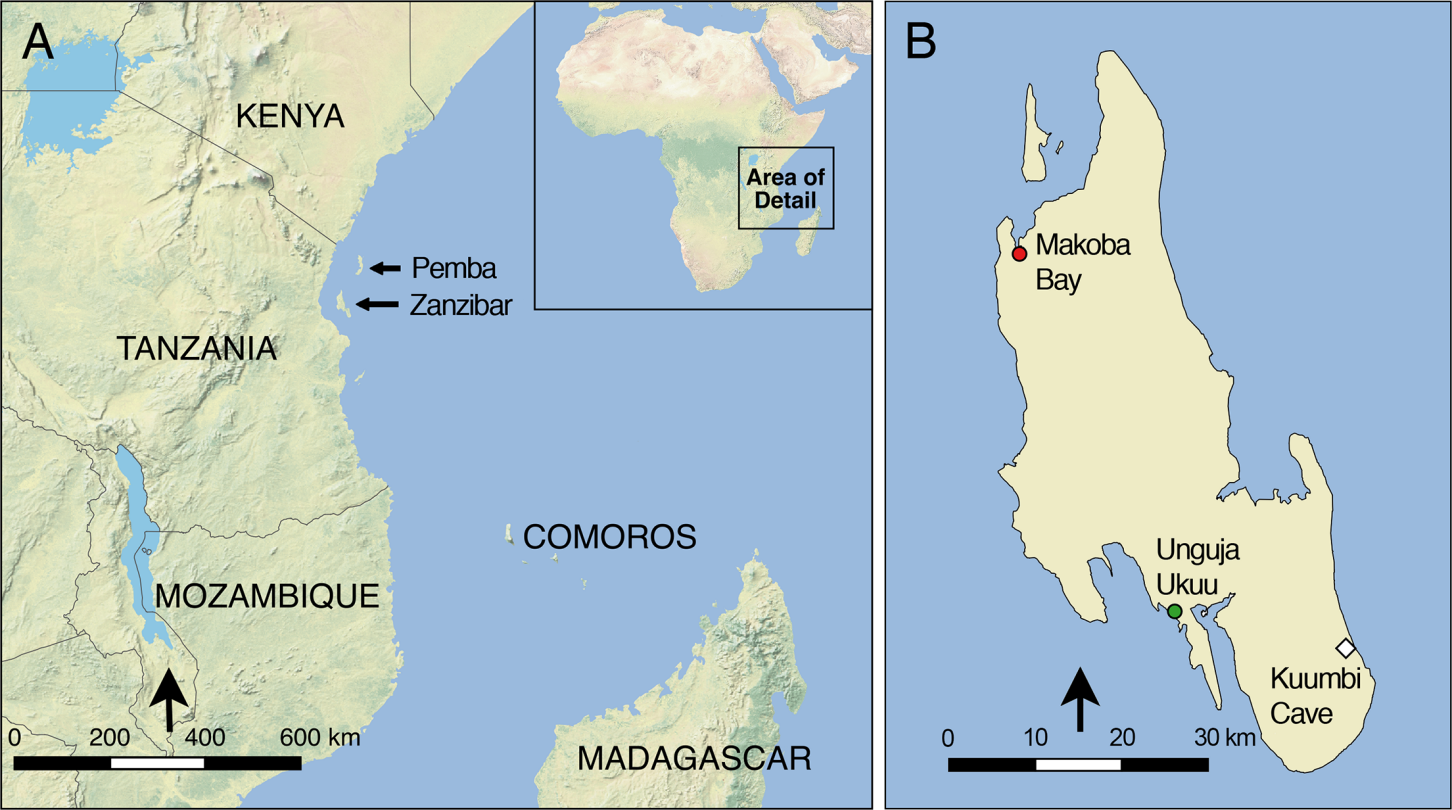
Location of sites discussed in text. A: eastern African coast, showing locations of Pemba and Zanzibar (Unguja) Islands. B: Zanzibar Island, with locations of coring and archaeological sites discussed in the text.

Zanzibar currently covers c. 1600 km^2^ and lies c. 40 km off the coast of Tanzania (Fig 1). It is separated from the mainland by a shallow channel (generally c. 30–60 m deep), which hosts a highly developed reef system [50–51]. Limestone reef terraces dominate the island, particularly in the southeastern area that is the focus of this study. This landscape includes typical karst features such as sinkholes and caves. The climate is characterized by wet/dry seasonality, with long rains from March to May and short rains from October to December. Vegetation belongs to the Northern Zanzibar-Inhambane coastal forest mosaic, which includes a mix of dry and moist forests, shrubland and grassland [52]. Today the Zanzibar vegetation is strongly shaped by agricultural activities, which have led to widespread clearing and the creation of protected forest and conservation “islands” such as the Muyuni and Jozani Reserves in the south and the Kiwengwa-Pongwe Reserve in the north. On the western side of the island are the deeper, fertile *kichanga* soils that have supported past and present farming communities, while the eastern area is dominated by thin and comparatively poor *kinongo* soils that overlie the coral rag; these soils support mainly low, scrubby bush. In the area immediately surrounding Kuumbi Cave, a taboo on tree-cutting due to the cave’s spiritual importance has preserved a small patch of tropical evergreen forest [48]. The island’s terrestrial mammals, described by Moreau and Pakenham [31–32], include three small bovids, monkeys, hyrax, giant pouched rat and small carnivores such as mongoose; the largest carnivore, the Zanzibar leopard (*Panthera pardus adersi*), was last documented in the late 20^th^ century but is now widely believed by zoologists to be extinct [53–54]. Many of the island’s most economically important wild species live offshore, in the rich reefs and backreef lagoons that have supported fishing communities during the past two millennia [55].

**Fig 2:**
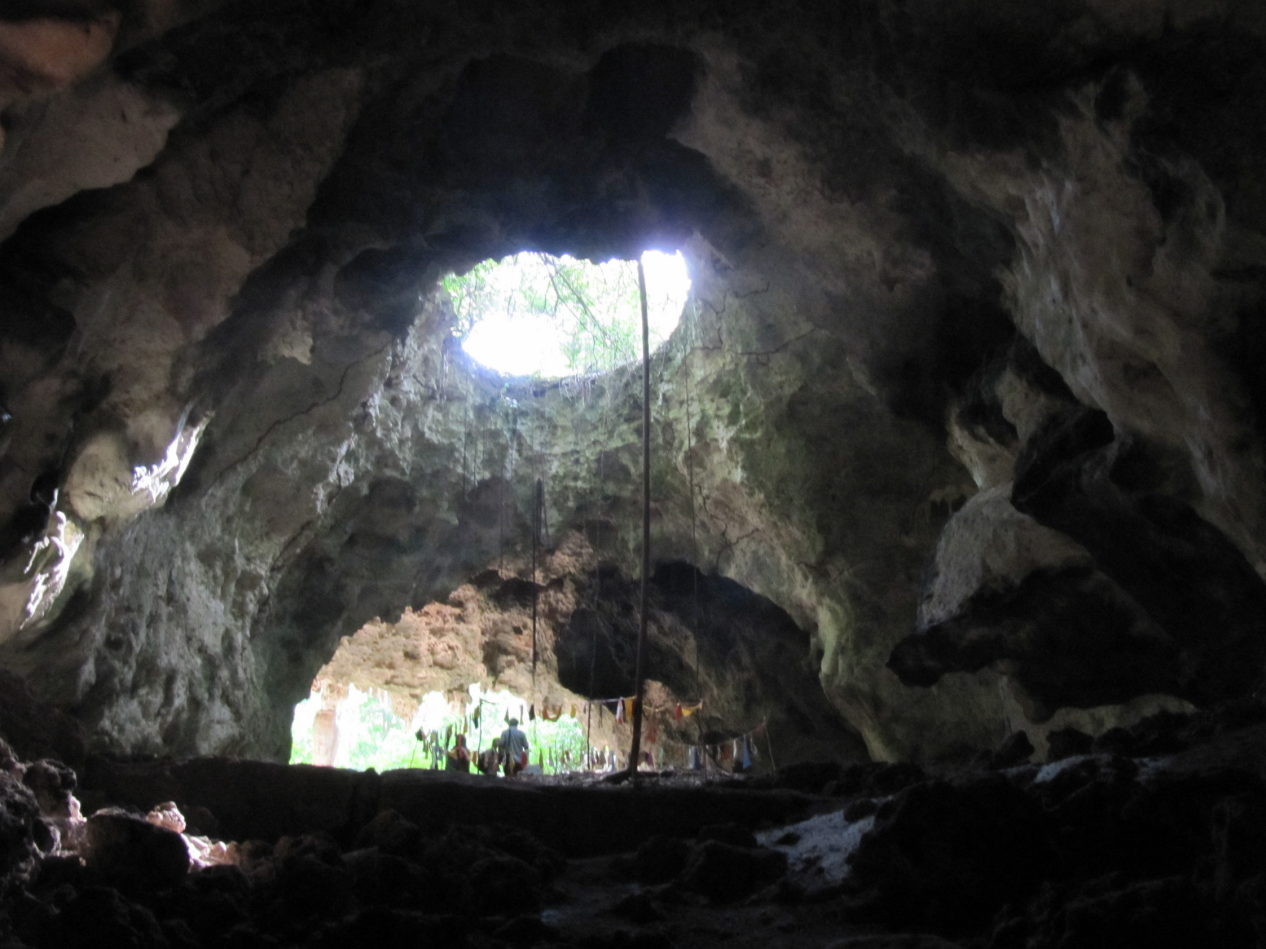
View of Kuumbi Cave. View of cave looking out from the largest cave chamber. Trench 10 of the 2012 excavation is located just behind the standing figures.

**Fig 3:**
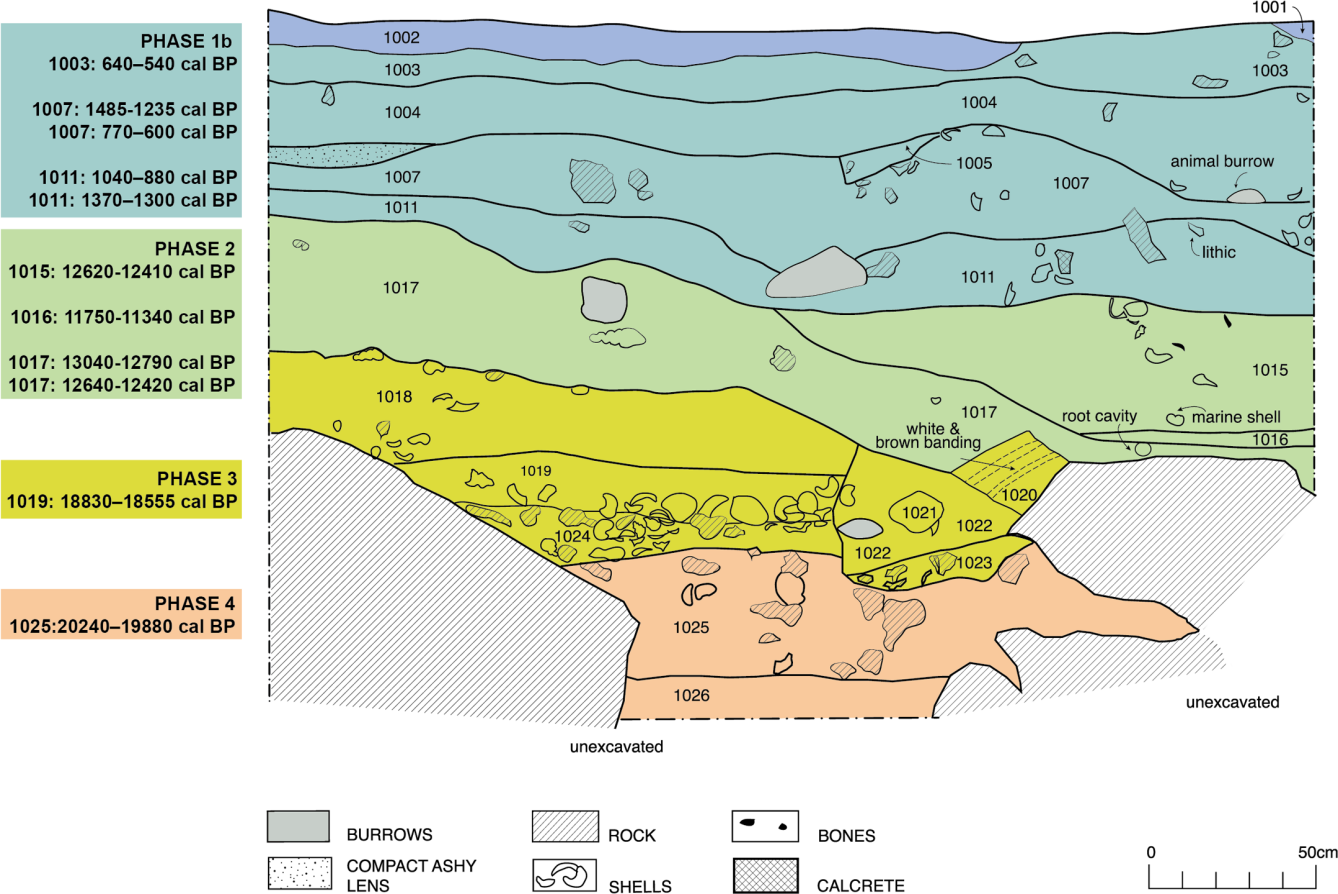
Kuumbi Cave Trench 10 stratigraphy. South section drawing based on [44], showing the occupational phases and calibrated dates before present (BP) that are linked to cultural events. See S1 Appendix for details of dating methods and materials, and other dates associated with sedimentary deposition.

Kuumbi Cave is located in Jambiani District, c. 2.5 km from the shoreline, and was never more than c. 7–8 km from the coast in the past 20,000 years, due to the steep continental shelf off the eastern coast of the island. It is a large solutional cave (Fig 2), one of several in a series of Pleistocene-era marine terraces on limestone [46]. The cave was excavated over multiple seasons [47–49], most recently through the 2012 Sealinks Project excavations described by Shipton and colleagues [44]. This campaign led to the identification of five phases in the main Trench 10 (3m^2^, 2.4 m depth), supported by a suite of 20 radiocarbon and optically stimulated luminescence (OSL) dates, as follows (Fig 3, S1 Appendix): Phase 1a (contexts 1001–1002) contains large limestone lithics and Swahili ceramics, and dates to the Later Iron Age (LIA, c. 1–0.5 ka). Phase 1b (contexts 1003–1013) contains the same lithics and earlier Tana Tradition/Triangular Incised Ware (TT/TIW) ceramics, dating to the Middle Iron Age (MIA, c. 1.35–1 ka). Phase 2 (contexts 1015–1017), by contrast, does not contain any ceramics but includes Later Stone Age (LSA) bone projectile points and other bone tools [45] and a quartz microlithic industry; this phase dates to the Pleistocene-Holocene transition at c. 13–11 ka. Phase 3 (contexts 1018–1024) contains similar LSA quartz and bone technologies and dates to the Late Pleistocene at c. 19–17 ka. Finally, Phase 4 (contexts 1025–1026) contains charcoal dated to c. 20 ka, and does not bear any unambiguous evidence of human occupation, but it does contain faunal remains, a few of which are burned.

Dating the Trench 10 sequence, discussed in detail by Shipton and colleagues [44], was complicated by two factors: animal burrowing and associated bioturbation, particularly in Phases 1 and 2, and a complex depositional sequence, particularly in Phase 2, that included colluvial inputs from upslope near the cave entrance, as well as from an opening in the ceiling. These factors likely explain the clear discrepancies between charcoal dates and those obtained directly on ceramics, shell or human bone (S1 Appendix). The latter set of dates showed a logical chronological progression, whereas many of the charcoal dates fell in the mid-Holocene and did not fit with associated archaeology. This is particularly true for Phases 2 and 1b, where mid-Holocene dates on charcoal were not replicated by any other dated samples. Based on geoarchaeological and chronometric findings, it was concluded that the dated charcoal from these phases derived from colluvial sediments containing mid-Holocene charcoal. By contrast, in the less disturbed Phases 3 and 4, charcoal dates were as expected, based on their stratigraphic relationship with dated land snail and marine shells.

This interpretation of the chronological and sedimentary evidence suggests that Kuumbi Cave was unoccupied throughout the Holocene until the MIA, challenging earlier interpretations of a food-producing “Neolithic” occupation of the cave as early as 6 ka [48]. Two other Zanzibar cave sites, Mwanampambe (undated) and Machaga (dated to c. 5 ka), have also been interpreted as having LSA and “Neolithic” occupations, respectively [48, 56]. However, stratigraphy and dating for Machaga are contested [57–58], and our new excavations at Kuumbi Cave demonstrate the problems with charcoal dates in complex cave sequences [44]. In light of the revised stratigraphy, it seems plausible to suggest that Zanzibar may have lost its human population during the Holocene after it became an island, in which case the new occupation in Phase 1b at Kuumbi Cave might be related to the emergence of the MIA open-air sites of Unguja Ukuu and Fukuchani [59–60] elsewhere on Zanzibar. These sites were settled by people with TT/TIW ceramics–demonstrating connections to the mainland and other offshore islands–and their occupants combined fishing and inshore foraging with crop cultivation, livestock-keeping, and hunting, as is common amongst contemporary Swahili-speaking islanders. However, without better-dated sequences from additional pre-Iron Age sites, it is impossible to rule out the possibility that Zanzibar–a large and archaeologically underexplored island–remained occupied by indigenous foragers until the arrival of mainland food producers.

## Separation of Zanzibar from the Mainland

Despite recent research activity in the region [40–42; 61], many questions remain about Holocene sea level change along the eastern African coast, and indeed across the entire southwestern Indian Ocean. Most studies in the southwestern Indian Ocean record an early Holocene sea level rise, but diverge on estimates of the amplitude and timing of the mid-Holocene highstand [62–63]. For example, studies of coral reef cores and terrace elevations in the Comoros, Mauritius and Réunion Islands do not indicate a mid-Holocene highstand [63–65], but suggest instead that after a rapid increase from 10–7.5 ka, sea level rise slowed until stabilizing at the present mean sea level (PMSL) around 3 ka. These findings broadly agree with those based on archaeological data from coastal eastern Africa, including Unguja Ukuu on Zanzibar [66], showing that locally the mid-Holocene highstand may have been followed by multiple drops to 0.5–1 m below PMSL during the last millennium, followed by a more recent rise. However, distinct patterns in the number, timing, and amplitude of highstands have been reported from other locations in Mozambique and South Africa [67–71]. Some of this regional complexity is almost certainly due to the complex pattern of interaction of glacio-isostatic processes following the last deglaciation, and possibly from tectonic movements of the Earth’s crust as well as changes in oceanographic conditions. Different proxies, error margins and scales of analysis can also render studies difficult to compare [72].

**Fig 4:**
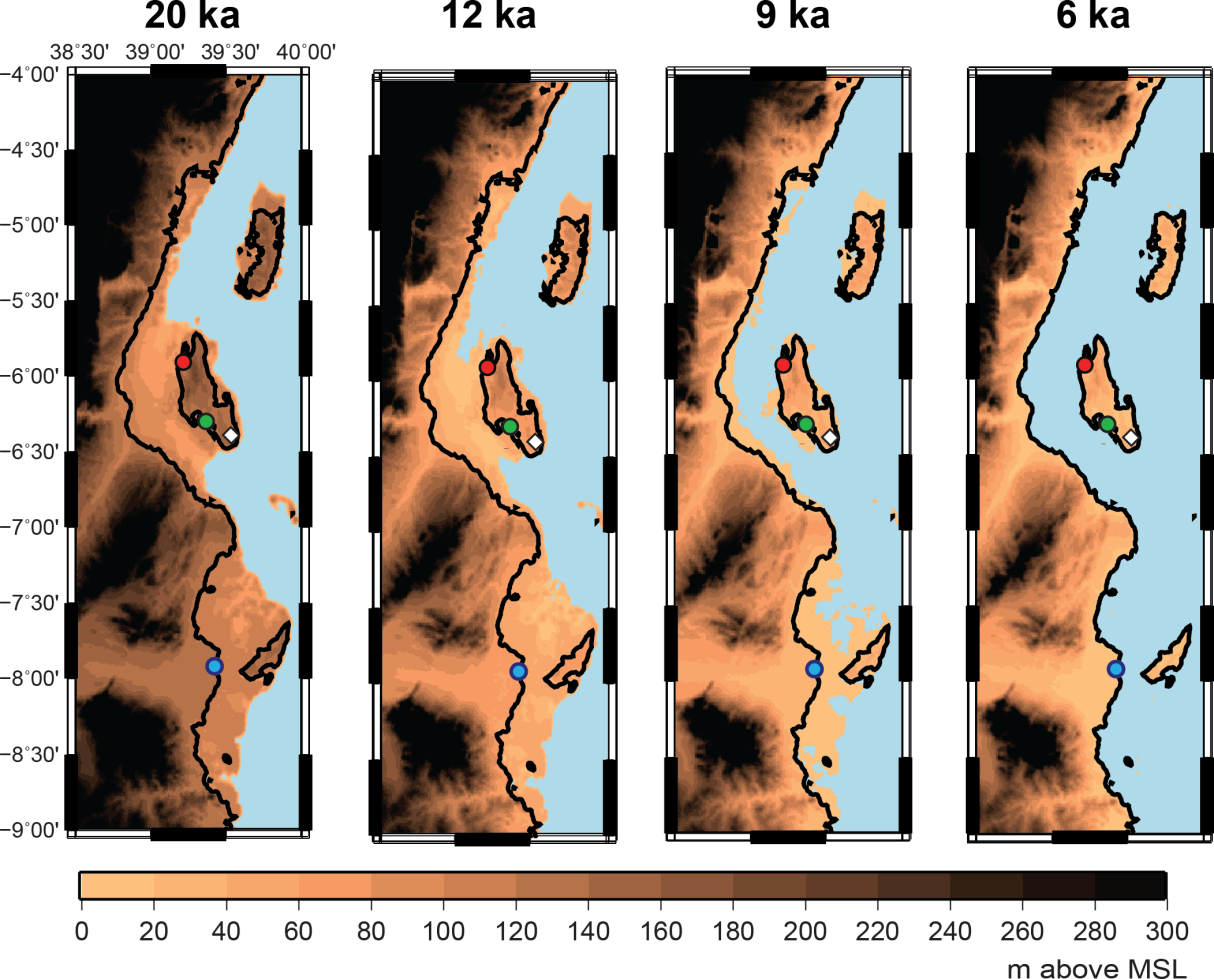
Reconstruction of northern Tanzanian coast 20–6 ka. Reconstruction at 20 ka, 12 ka, 9 ka, and 6 ka of the topography of the northern Tanzanian coast in meters above Mean Sea Level (MSL) at the time under consideration, with the outline of the present-day coastline. The three largest islands are, from north to south, Pemba, Zanzibar (Unguja), and Mafia. Circles mark coring locations: red, Makoba Bay; green, Unguja Ukuu; blue, Rufiji Delta. The white diamond marks Kuumbi Cave.

**Fig 5:**
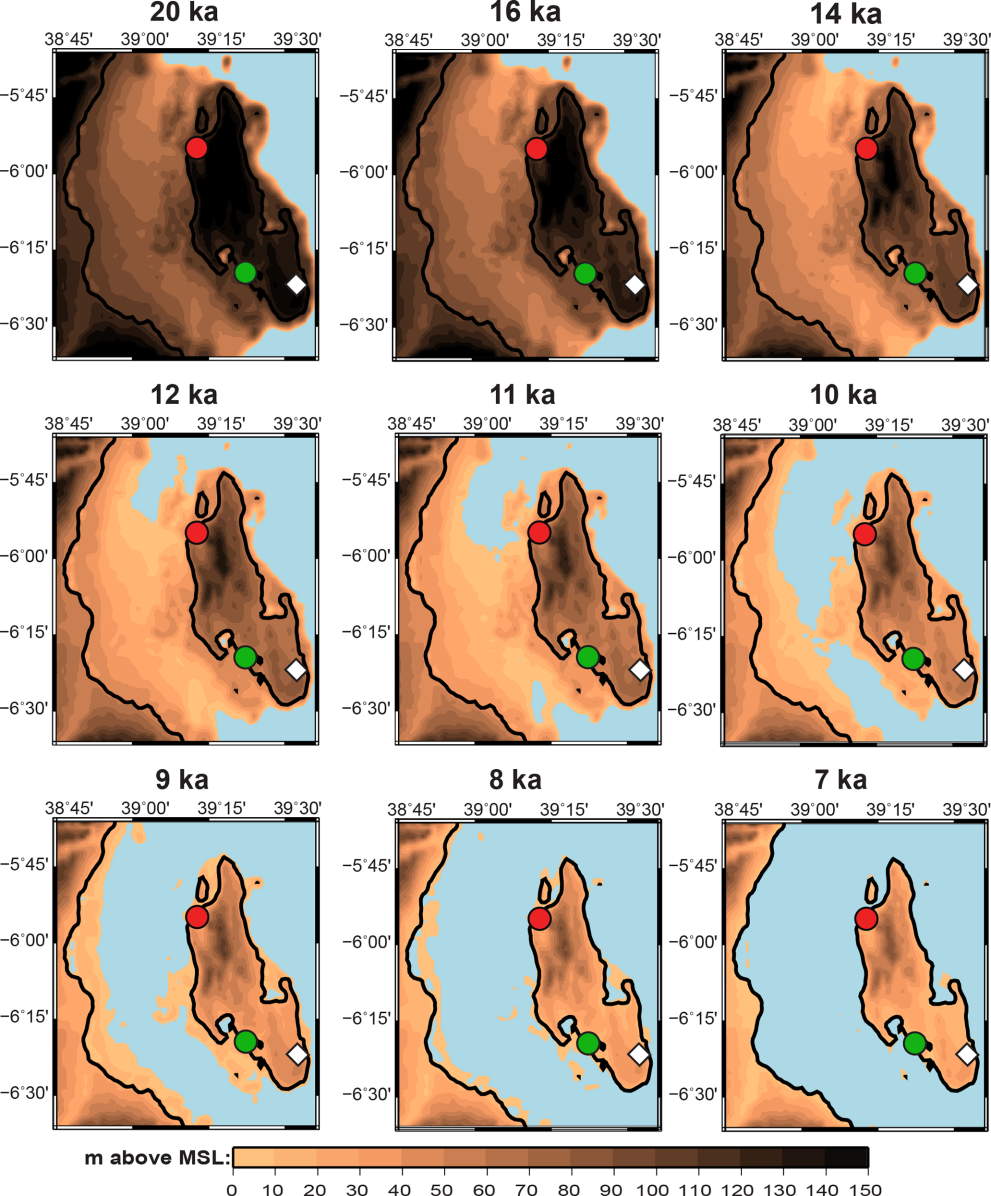
Reconstruction of Zanzibar 20–7 ka. Reconstruction of the topography of Zanzibar and neighboring Tanzanian mainland in meters above Mean Sea Level (MSL) at the time under consideration, with the outline of the present-day coastline. Circles indicate coring locations: red, Makoba Bay; green, Unguja Ukuu. The white diamond marks Kuumbi Cave. Note that the scale is not identical to that in Fig 4.

Given this regional variability, and the need for chronological resolution to link the tempo of island formation to local archaeological sites, we developed a reconstruction of the formation of Zanzibar and the neighboring coast that is based on both modeling and local observational data. We predicted the evolution of relative sea level (RSL; relative to PMSL) using a model described elsewhere [73–74]. This observationally-constrained model calculates the Earth and ocean deformation response to the build-up and retreat of ice sheets during glacial cycles, taking into account changes in gravity, the migration of shorelines, and changes in seafloor bathymetry. The model has been calibrated for the past 20 ka from a global database that includes the western Indian Ocean [75]. Our results (Figs 4 and 5) illustrate the shaping of the northern Tanzanian coastline over the past 20 ka, and demonstrate the speed with which Zanzibar became isolated c. 12–9 ka.

**Fig 6:**
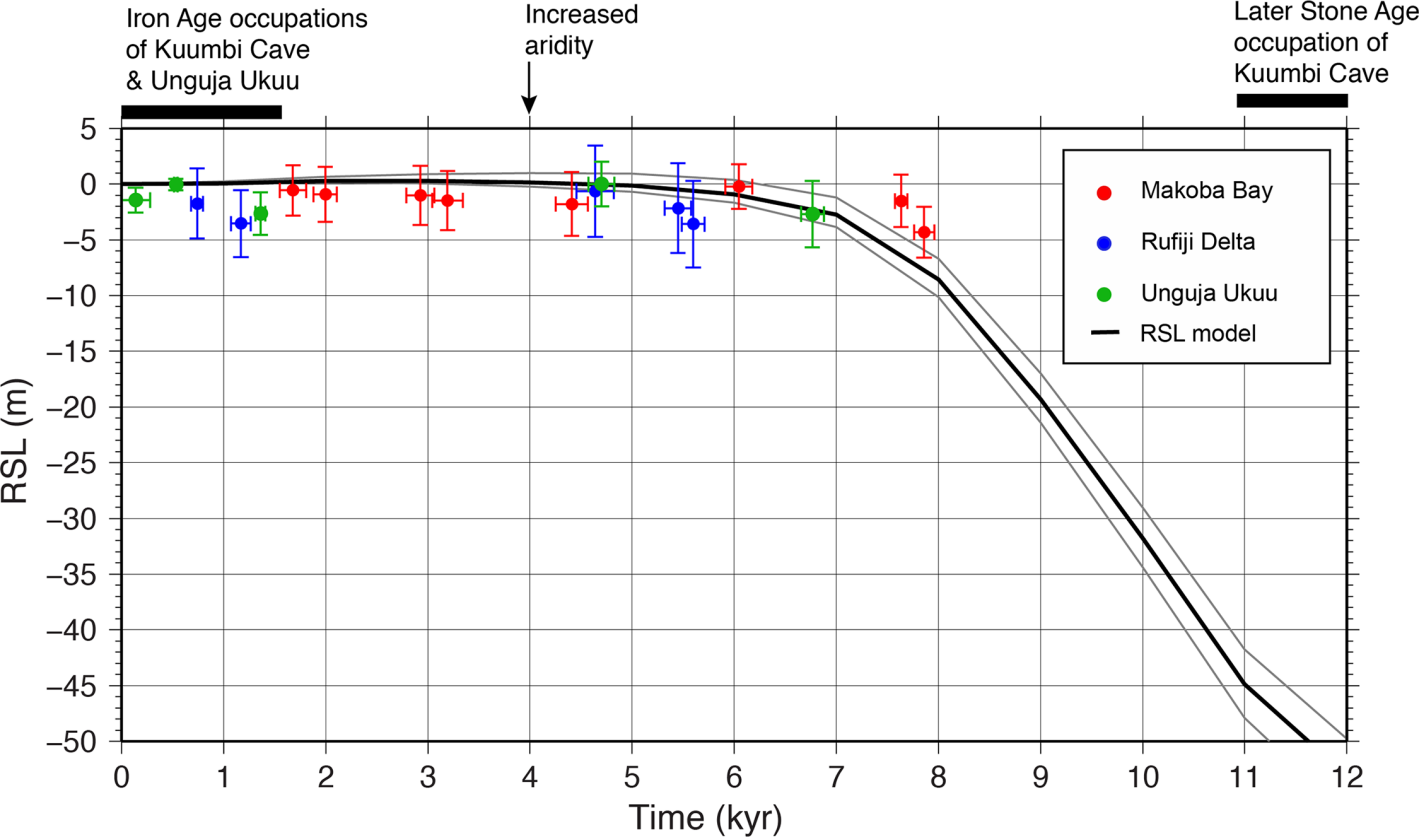
Comparison of model and observational data for relative sea level (RSL). Comparison of RSL predictions and Holocene RSL observational evidence obtained from sediments on three mangrove sites in Zanzibar and mainland Tanzania [40–42; 61] with occupation timeline of Kuumbi Cave indicated. See Methods for explanation of error margins.

These predictions are compared to recent field data from Zanzibar and the Tanzanian coast. Paleoenvironmental and paleoecological data (stratigraphy, pollen, charcoal and loss on ignition) were obtained from seven sediment cores from mangrove areas in the northwest (Makoba Bay) and southwest (Unguja Ukuu settlement) of Zanzibar and from three cores in the northern Rufiji Delta [40–42; 61]. These mangrove observational data largely agree with the modeled RSL predictions (Fig 6). Discrepancies, particularly around 8 ka, might be due to a local effect, or to underestimation of the model and observational margins of error (see Methods). The two observational points from Makoba Bay at 8 ka that mark the beginning of mangrove formation date to the end of the rapid phase of deglaciation, when sea level rose at c. 12m/ka [75]. In the absence of earlier sedimentary records we can only say that these points are consistent with the RSL prediction of rapid submergence of the Zanzibar channel beginning between 12–11 ka, and the eventual isolation of Zanzibar by 9–8 ka.

Pollen records recovered from sediment cores at Makoba Bay, the Unguja Ukuu settlement, and the Rufiji Delta indicate changing mangrove compositions, suggesting a local signal of lower RSL around 4 ka. This is coincident with–though not necessarily causally linked to–a regional drought phase that has been documented across eastern Africa [76–77]. This mid-Holocene drought has been recorded in the opening up of savannas [78] and lowering of lake levels; isotopic data at Lake Malawi show an increase in C_4pot_ vegetation around 4 ka [79]. Although not of the same magnitude, paleoenvironmental proxies on the mainland record a variable environment during the late part of the Holocene [78], while mangrove records indicate a period of relative stability in sea level [40–42]. The next major recorded change from the Unguja Ukuu sediments occurs at around 500 BP, when there is a massive increase in charcoal input thought to relate to Portuguese arrival on the island and an Islamic reoccupation of Unguja Ukuu, events that led to substantial human impact on mangrove ecosystems. Although a recent sea-level reconstruction based on mangrove composition at Unguja Ukuu is complicated by these anthropogenic impacts, at Makoba Bay (located some 20 km to the north) there is no discernable human impact and there is a record of recent minor RSL rise [80].

## Diachronic Changes in Faunal Diversity at Kuumbi Cave

**Fig 7:**
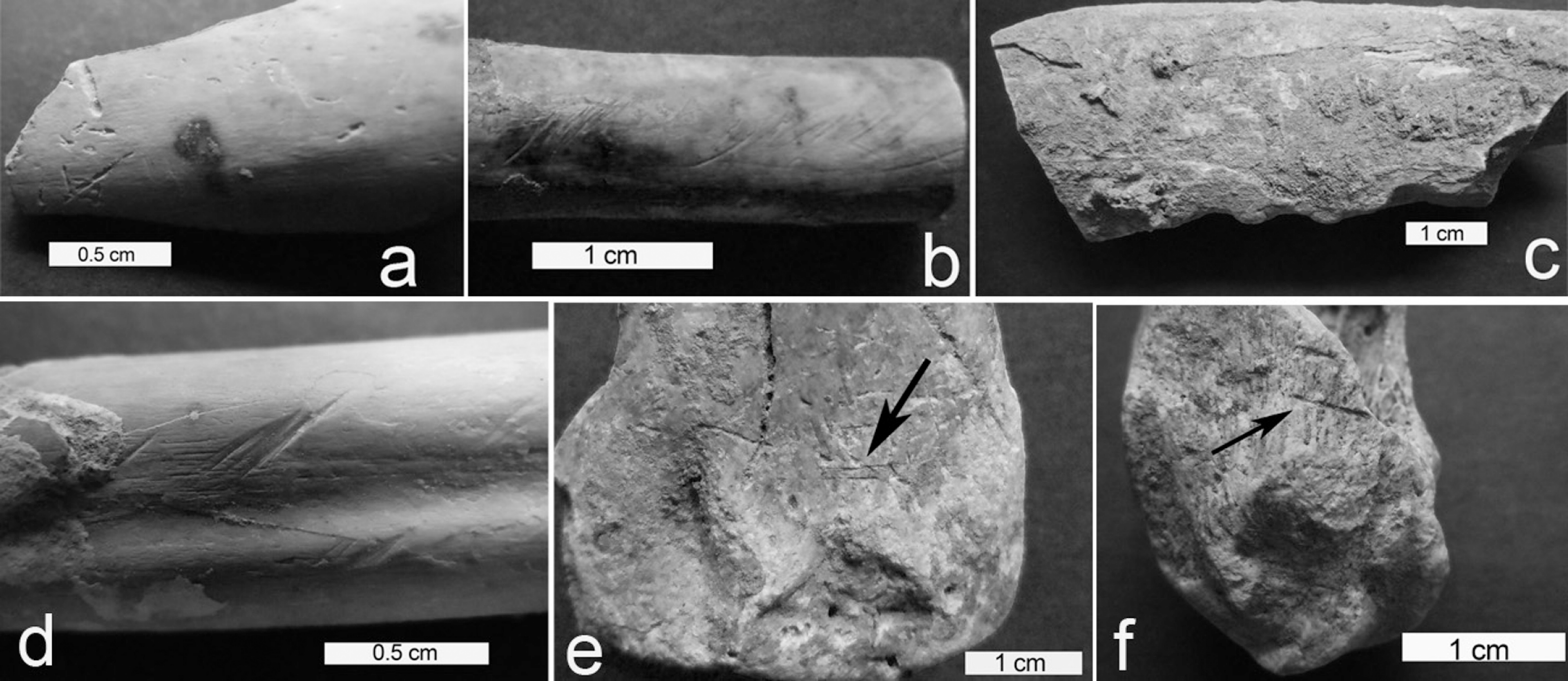
Bone surface modifications in Kuumbi Cave Trench 10. Examples of bone surface modifications in the Trench 10 assemblage: a, biochemical pitting from soil or root bacteria; b, shallow, parallel oblique lines indicating sedimentary abrasion; c, micro-notches along the breakage plane, likely produced by a carnivore; d, overlapping cut marks and abrasion on limb bone of duiker; e-f, cut marks on limb bones of reedbuck and bushbuck, respectively.

This modeled reconstruction of island formation provides a useful framework in which to interpret the faunal assemblage at Kuumbi Cave. Analysis of the fauna from the Trench 10 excavations resulted in the identification of 117 fish specimens (Number of Identified Specimens, NISP), a minimum of 3,556 individuals (Minimum Number of Individuals, MNI) of terrestrial and marine molluscs, and 6,667 tetrapod NISP. Descriptions of the fish and mollusc assemblages are reported elsewhere [44]. A detailed taphonomic analysis of 5,118 tetrapod NISP from selected contexts, including numerous limb bone shafts, shows that many biogenic and anthropogenic processes affected the assemblage (Fig 7, S2 Appendix). Sedimentary abrasion affected 1% of specimens, sometimes co-occurring with cut marks, which if shallow can be difficult to distinguish from the former. A conservative approach was taken, with definitive cut marks identified on just 1% of the bone assemblage, rising to 2% when excluding specimens with poor cortical visibility, and to 4% when focusing on larger-bodied fauna that would have required more butchery. Despite this low human signal, the dominance of green fractures (71% of observed limb shafts) indicates that bones were mainly broken while fresh. The dearth of carnivore tooth pits (<1% of bone specimens) or notches with typical carnivore morphology (<1% of limb shafts), and the relatively high frequency of burnt bone (14% overall, rising to 24% in Phase 2), imply that humans were the primary bone-breaking agent, and are mainly responsible for the accumulated fauna, particularly in the case of larger prey taxa.

Since most of the macrofaunal assemblage appears to be anthropogenic, we can interpret the faunal spectrum in terms of available prey and of human choices during the Late Pleistocene, terminal Pleistocene, and late Holocene, enabling an analysis of diachronic change (S1 Table). Smaller prey taxa remain remarkably consistent throughout the Trench 10 sequence, with the three dwarf bovids of modern-day Zanzibar being the dominant taxa during all phases: blue duiker (*Cephalophus monticola*), Ader’s duiker (*Cephalophus adersi*) and suni (*Neotragus moschatus*). Also present in all phases are bushpig (*Potamochoerus larvatus*), tree hyrax (*Dendrohyrax validus*), giant pouched rat (*Cricetomys gambianus*), and colobus (*Colobus* sp.) and cercopithecine monkeys (*Cercopithecus* or *Chlorocebus* spp.). While hyraxes and monkeys are common in the cave and surrounding forest today, at least some of them entered the assemblage as food remains, as inferred from cut marks. Other taxa also tend to inhabit or frequent caves, and are suspected to be unrelated to human occupation at Kuumbi Cave; these include leopard (*Panthera pardus*), mongooses (Herpestidae), civets or genets (Viverridae), murid rodents (Muridae), elephant shrews (Macroscelididae), snakes (Serpentes), and fruit bats (Pteropodidae). However, none of these can be entirely ruled out as potential food species, as demonstrated by Walsh’s [28] description of bat-eating on Pemba.

**Fig 8:**
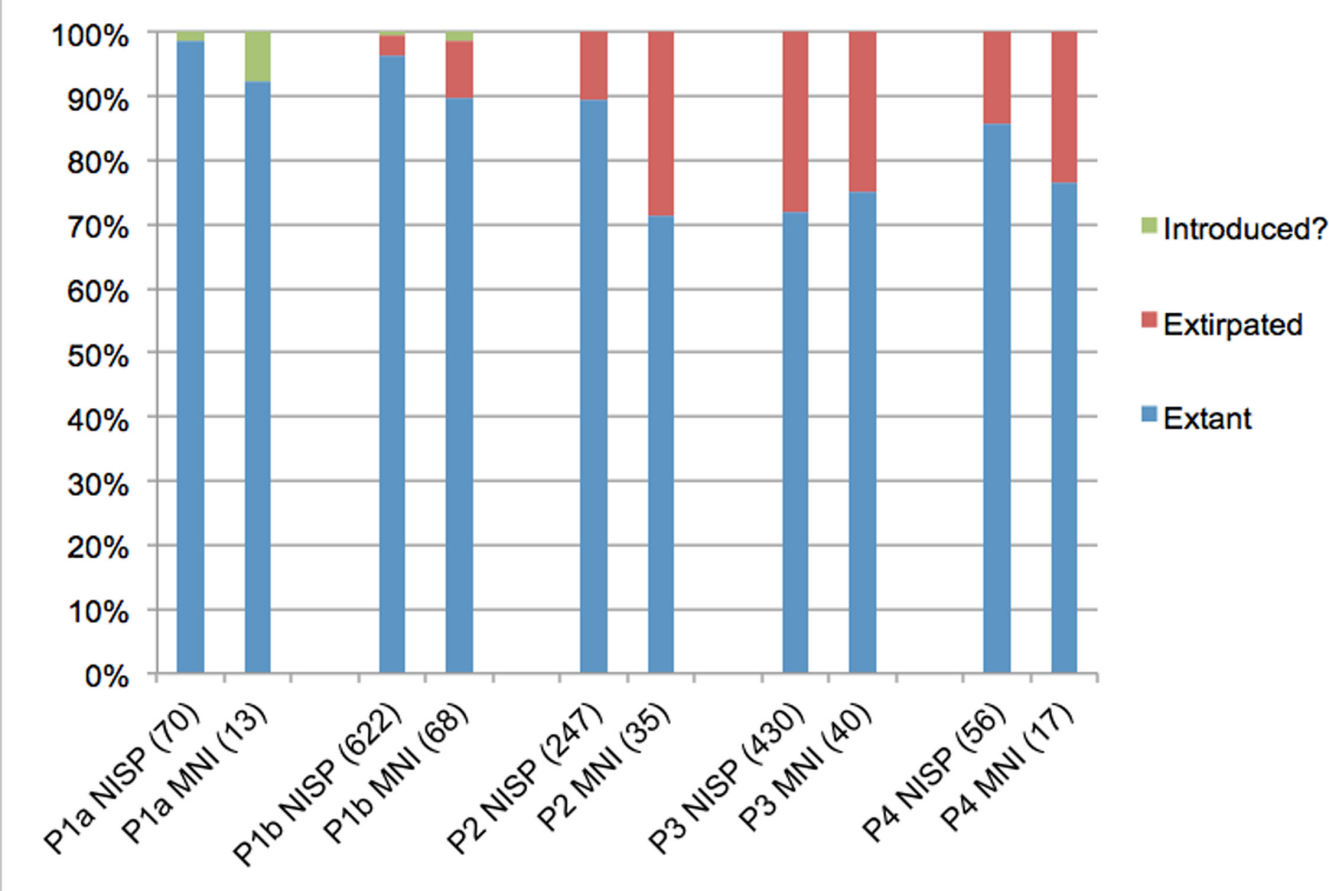
Extirpated, extant and introduced fauna in Kuumbi Cave Trench 10. Relative frequency of nonhuman mammalian fauna that are extant or extirpated on Zanzibar today. “Introduced?” are remains of possible domestic cattle; we stress the tentative nature of this identification, given the paucity of diagnostic elements and the possible persistence of similarly-sized extirpated bovids. Extirpated fauna include: zebra, steenbok, bush duiker, bushbuck, reedbuck, waterbuck, buffalo, white-tailed mongoose, hare, porcupine, and a possible Thomson’s gazelle. Microfauna and remains only identifiable to size class (e.g., “Mammal Size 2”) were excluded from this analysis. NISP, number of identified specimens; MNI, minimum number of individuals.

**Fig 9:**
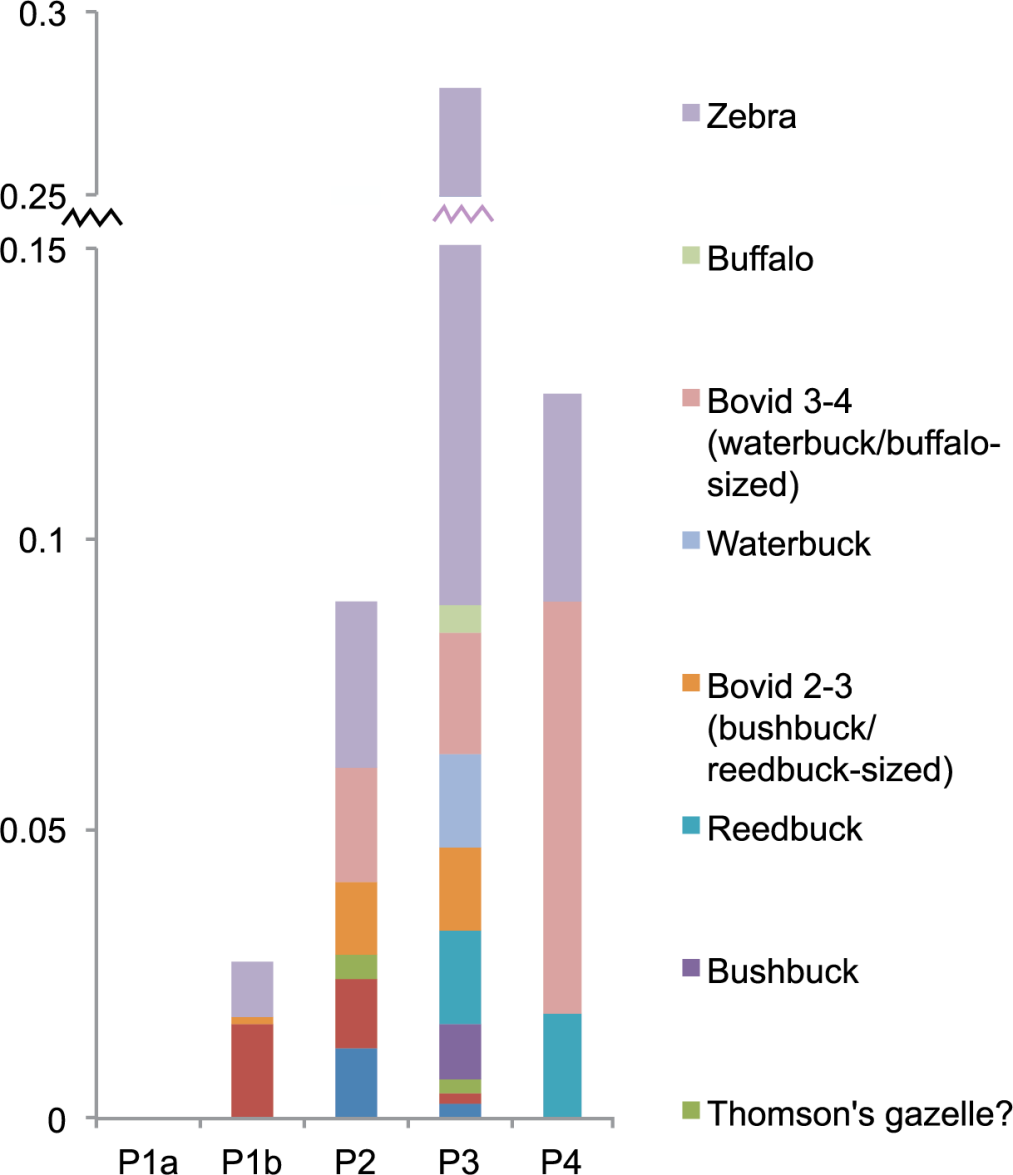
Extirpated bovids and equids in Kuumbi Cave Trench 10. Diachronic trends for extirpated bovids and equids only, with frequencies for these taxa expressed as a percentage of total NISP (number of identified specimens) for all fauna in each phase. For simplicity, tentative identifications to taxon were treated as if they were positive; for example, Cf. *T*. *scriptus* and *T*. *scriptus* are both listed here as bushbuck. For detailed data, see S1 Table.

**Fig 10:**
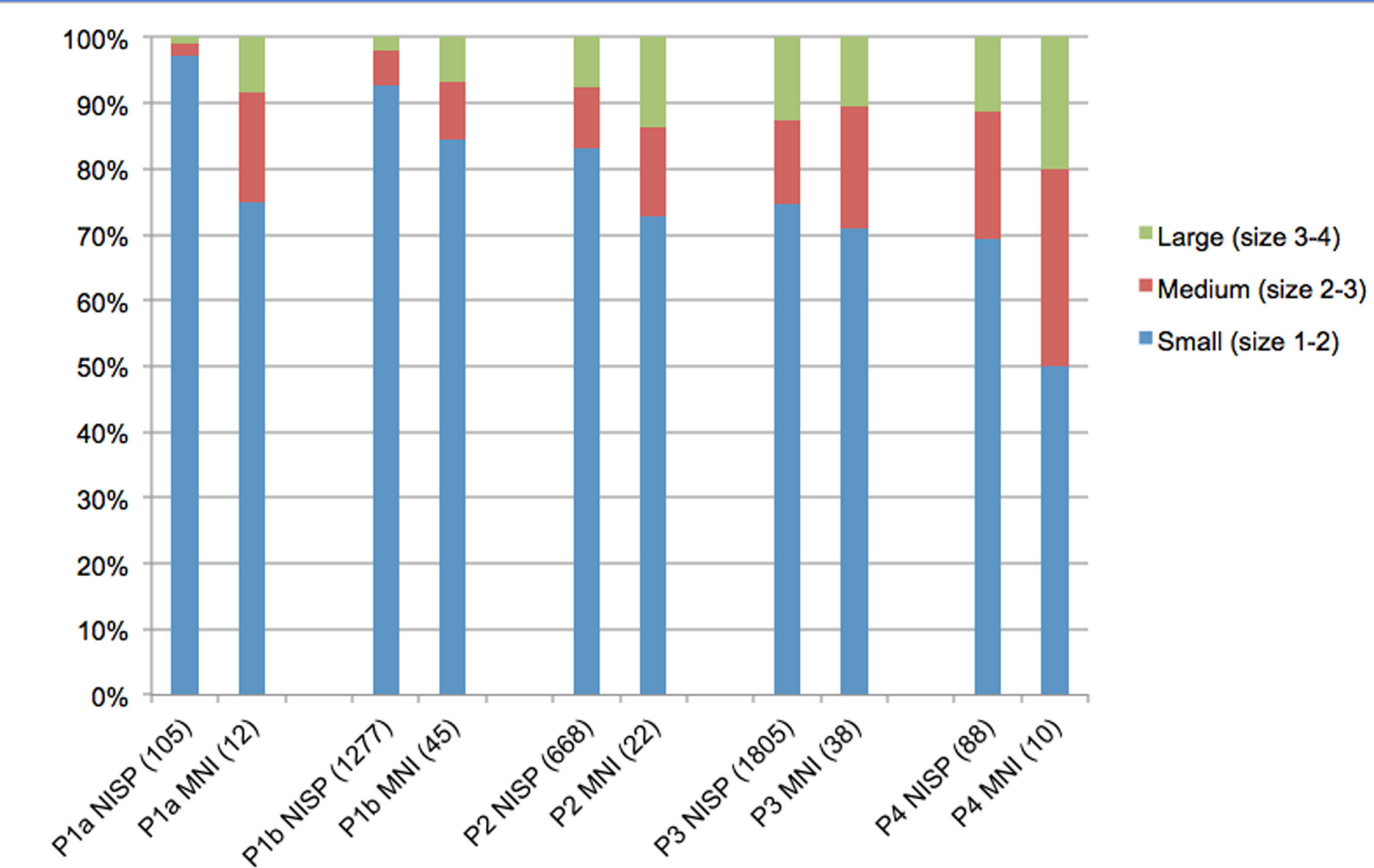
Decline in large mammals in Kuumbi Cave Trench 10. Diachronic trends in nonhuman mammalian carcass sizes (see Methods), excluding mammals smaller than Size 1 (e.g., rodents, bats, hyrax, mongoose, galago). Some of the medium and large animals in Phase 1 are cattle- and caprine-sized bovids but could not be identified to taxon, and might be either domestic or extirpated taxa. NISP, number of identified specimens; MNI, minimum number of individuals.

While the above fauna are present on Zanzibar today, the Trench 10 sequence attests to the disappearance of numerous other taxa after island formation. In the Late and terminal Pleistocene (Phases 4–2), large fauna were present, particularly zebra (*Equus* cf. *quagga*), which, based on their size, were likely plains zebra. Also present were buffalo (*Syncerus caffer*), waterbuck (*Kobus defassa*), reedbuck (*Redunca redunca*) and bushbuck (*Tragelaphus scriptus*). Breakage patterns and cut marks in Phases 3–2 indicate that these fauna were brought to the cave by humans, not carnivores, despite the minor presence of leopard in the site. Some of the fauna–especially zebra and possible Thomson’s gazelle (*Gazella thomsoni*)–are indicators of a relatively open environment, while other bovids present thrive in mixed and closed habitats. None of these fauna are present on Zanzibar today, nor are some of the smaller bovids identified, such as steenbok (*Raphicerus campestris*) and bush duiker (*Sylvicapra grimmia*) [28, 32]. Some small fauna found in low numbers in the terminal Pleistocene phase, such as porcupine (*Hystrix cristata*) and hare (*Lepus* sp.), are also not documented on Zanzibar today. Figs 8 and 9 illustrate clear diachronic trends in which presently extirpated fauna, which make up approximately 10–30% of the Phases 2–4 assemblages, become less common through the sequence as the assemblage becomes increasingly dominated by duiker-sized bovids. Even among these smaller bovids, however, some taxa disappear by the final Phase 1a, including bush duiker, common in Phases 1b-3, and steenbok, found in Phases 2 and 3. By Phase 1a, only locally extant fauna are identified, though a small number of specimens from medium and large bovids (Sizes 2–3) were not sufficiently diagnostic to enable distinction between domestic and extirpated bovids. These exceptions aside, there are clear trends in the decreasing abundance of large and medium-sized taxa after the terminal Pleistocene, and in the persistence of select smaller-bodied taxa over time (Fig 10), with the assemblage decreasing in richness over the course of the Holocene (S1 Fig). This is perhaps clearest in the example of the zebra, which is found in abundance in Phase 3 but in decreasing numbers in Phases 2 and 1b, and disappears entirely by Phase 1a (S1 Table).

Our findings are distinct from those reported for previous excavations. The extirpated fauna reported by the earlier campaign [48] include elephant, giraffe, equid, buffalo, reedbuck, steenbok, dik-dik, baboon, and jackal, as well as crocodile, which is reported as an occasional “vagrant” on Zanzibar today but is normally absent [33]. Only equid, buffalo, reedbuck and steenbok are reported by both analyses. The contrast between these reports can be interpreted in two ways. First, the previously excavated [48] faunal assemblage (tetrapod NISP = 3,728 from six trenches, excluding human remains) comes from a much greater sediment volume (>40 m^3^) than our own (7.2 m^3^), and therefore it can be expected that a wider range of taxa might be present. Indeed, the faunal spectrum of Zanzibar prior to its isolation must have included other mammals, in addition to birds, reptiles and microfauna not identified at Kuumbi Cave. This underscores the need for additional research from other sites on Zanzibar with substantial time-depth. A second issue is that some fauna from the prior excavations may have been misidentified. A notable example is that 13% of tetrapod, nonhuman NISP in the six trenches reported by the earlier campaign [48] are domesticates, including cattle (*Bos taurus*), caprines (goat, *Capra hircus* or sheep, *Ovis aries*), dog (*Canis familiaris*), chicken (*Gallus gallus*), and either wild or domestic cat (*Felis* sp.). By contrast, in Trench 10, <1% of NISP identified to taxon are possible cattle, all recovered from Phase 1, and no other domesticates are identified. This absence of domesticates is consistent with evidence from other cave deposits, for example in the Mafia archipelago [81] and the southern Kenyan coastal hinterland [82], where, despite the presence of Iron Age material culture, wild fauna remain the focus of subsistence.

## Discussion

The Kuumbi Cave faunal data suggest that the extirpation of numerous mainland taxa on Zanzibar Island took place between the terminal Pleistocene and late Holocene. This site bears the only record of wild mammals larger than duiker on the eastern African islands (aside from Madagascar), and is the only witness to their disappearance from Zanzibar. Five securely-dated phases at Kuumbi Cave predate, span, and postdate the period of Zanzibar’s most recent isolation from the mainland, as modeled here from sea level data for the first time. Faunal data from these windows indicate that defaunation occurred after island formation, and suggest that most of the now-extirpated fauna were sharply reduced or perhaps eliminated prior to the MIA. However, the Kuumbi Cave sequence also provides clear indication that some large animals, most notably zebra, were not extirpated until after the arrival of food producers from the mainland, as demonstrated by their persistence in Phase 1b. This persistence is notable, given the zebra’s typically large home range and grassland requirements. It is also remarkable that the island’s apex predator, the leopard, present in Phases 4 to 1b, survived into the twentieth century, though it may have been less vulnerable to prey loss if it began to feed on domestic livestock. These findings call for more detailed exploration of the island’s trophic dynamics from additional datasets.

These findings make Zanzibar unique among African continental islands. The only other African islands with archaeological records possibly predating island formation, Bioko and Elobey in Equatorial Guinea [83–84], have neither firm dates nor published faunal data. Comparisons to oceanic islands such as Mauritius and Seychelles, or to the island-continent of Madagascar, are less appropriate as these hosted unique endemic fauna whose disappearance, in many cases, has been unambiguously linked to human occupation [18, 22, 85–86]. Therefore, comparative case studies must be sought outside African contexts, for example in the post-LGM breakup of Santarosae Island into some of the present-day Californian Channel Islands. Decades of research on these islands have illuminated the relative contributions of geographic isolation and human occupation to defaunation, e.g., [87–88; 15]. These studies demonstrate that in some cases–such as that of mammoths–humans apparently played no role in local extinctions, while in other instances, humans had major impacts through their foraging activities and translocations of nonnative plant and animal species. Similarly, a study of faunal reductions on Tobago [14] demonstrates the difficulty in teasing apart human and biogeographic influences; while these are not mutually exclusive, the authors show that detailed faunal records can indicate which taxa disappear and when, a key starting point in the study of island defaunation. We can expect that with further research, a more sophisticated picture may emerge of Zanzibar’s biotic and human history. On present evidence, we can suggest different scenarios as to what may have happened as sea levels rose rapidly following the LGM, hypotheses that can be tested with additional well-dated archaeological and faunal assemblages from Zanzibar.

One possibility is that as the process of isolation began c. 12 ka, with crossings perhaps possible only through a shallow strait by c. 9 ka, faunal relaxation did occur, at least partially. Relaxation initially affects fauna that require a larger home range [89], in this case zebra, buffalo and waterbuck. As these highly-ranked prey declined in number, humans likely continued to exploit smaller animals, but could also have expanded their diet to include lower-ranked prey and more marine resources, possibly moving away from sites like Kuumbi Cave and toward the mangroves and the expanding shoreline on the western side of the nascent island. The narrow land-bridge that extended between Zanzibar and the mainland until c. 9 ka according to our modeled reconstruction would have provided access to extensive shallows rich in marine resources, and may have attracted foragers to move from the island to the faunally richer mainland. It may be that Zanzibar was abandoned at this time, or that a small remaining population of foragers persisted, or depopulated and repopulated the island multiple times in subsequent periods, without leaving any trace in Kuumbi Cave’s discontinuous sequence. If occupation did shift away from the forest and toward the coast, many of the resulting sites would have later been lost to the sea as it continued to rise until c. 6 ka, and these sites would now only be discoverable through underwater exploration. Indeed, this may be the reason that there are few coastal sites in eastern Africa for the Late Pleistocene-Holocene that might offer useful comparative data with which to evaluate the hypothesis of intensified coastal resource use [90].

The known sequence at Kuumbi Cave suggests that Zanzibar was abandoned or depopulated in the early Holocene and only reoccupied in numbers during the late Holocene Iron Age, with the expansion of Bantu-speaking mixed farmers onto the eastern African islands and the occupation of coastal settlements such as Unguja Ukuu and Fukuchani [60]. However the faunal assemblage in Phase 1 has high taxonomic diversity including many non-ungulates; this phase also contains the bulk of Trench 10’s fish remains (mainly reef fish) and shows an increased emphasis on shellfish collection [44]. This diversity suggests that Kuumbi Cave may have been occupied by full-time foragers during the late Holocene, based on contrasts with ethnographic data concerning food producers’ hunting preferences [91]. These foragers could either have been descendants of indigenous island populations, or new seafaring arrivals from the mainland (possibly responding to the changes wrought by food production). In either case, these foragers’ contact with food producers on the coast might explain the uptick in marine resources, the ceramic assemblages, and the few remains of possible cattle in Phase 1. As noted earlier, evidence from Mwanampambe and Machaga Caves, along with previous interpretations of the Kuumbi Cave stratigraphy, have led to claims of LSA foraging and “Neolithic” food producing occupations on Zanzibar in the middle Holocene, c. 5–2 ka [48, 56]. Our recent excavations at Kuumbi Cave, supported by a large radiocarbon dataset, did not corroborate this scenario [44]. However, given the island’s large size and the lack of secure dates from other sites, the possibility of a pre-Iron Age foraging occupation, or even multiple reoccupations–though not observed at Kuumbi Cave–cannot be ruled out. If the island were continually occupied by foragers, even greater weight could be given to human agency in Holocene-era defaunation.

Regardless of this unresolved issue, it is clear that some fauna present during the MIA at Kuumbi Cave–such as zebra and bush duiker–are absent today. This implies that while populations of now-extirpated fauna may have been reduced over the course of the Holocene to the point where they could no longer replenish themselves, human activities within the past two millennia may have dealt the final blow. Faunal relaxation and human agency might well have worked in tandem to produce the drop in species richness observed in the Trench 10 assemblage: populations of large fauna might have been reduced by environmental isolation during the Holocene to the point where they could not withstand new pressures associated with the arrival of farmers in the MIA. These pressures would have included the clearance of natural vegetation for settlement and agriculture, and the translocation of domestic and commensal species such as goat and black rat (*Rattus rattus*), as recorded at MIA sites like Unguja Ukuu [59]. These introduced taxa have been shown in other island settings to have had major impacts on indigenous flora and fauna [92–95]. It can be presumed that this may also have happened on Zanzibar, though the details remain to be discovered through study of additional sedimentary, paleobotanical and faunal records. MIA populations on Zanzibar also hunted, as documented at the sites of Unguja Ukuu and Fukuchani [59–60]. While they focused mainly on extant small bovids, presumably for food, they may also have killed animals for products such as skins, or in order to protect their homes and crops. These activities would have had a significant effect on island biota.

## Conclusions

Kuumbi Cave is the earliest published archaeological site for the eastern African coast and islands, and has produced an unparalleled faunal record, supported by detailed geoarchaeological and chronometric analyses. Combining this record with the first systematic analysis of Zanzibar’s formation, with rising terminal Pleistocene to early Holocene sea levels, has enabled us to examine patterns of defaunation on the island in relation to both isolation and human activity. While human impacts to some fauna appear highly likely, the power of this analysis has been to provide a set of hypotheses about defaunation that can be tested against further archaeological and paleoecological datasets.

Our data show that faunal diversity at Kuumbi Cave declined over the course of the Holocene, and we present alternative scenarios concerning whether or not the island was occupied at this time. The question of occupational continuity, which cannot be established without further systematic archaeological work at a broader range of Zanzibar sites, has important implications for assessing the relative impacts of island biogeography and human agency on processes of defaunation. What our data do clearly show is that when mainland food producers arrived on Zanzibar, faunal diversity on the island was greater than at present, suggesting the plausibility of an anthropogenic contribution to the final extirpation of zebra and some bovids. Based on currently available data, Zanzibar appears to be unique among African continental islands in that it hosted such populations of large mammals, adapted to a wide range of both open (e.g., zebra, buffalo) and closed (e.g., waterbuck, reedbuck) environments, for several millennia before their extirpation. On present evidence, these fauna appear not to have evolved (for example, by becoming smaller) in response to the island environment; however, island conditions did produce several endemic species that are distinct from mainland populations, such as the Zanzibar leopard and red colobus monkey. These endemics survived hunting and habitat destruction for several millennia, again making Zanzibar distinct from other continental islands of Africa. These findings contribute to a growing body of research on the Anthropocene, and its potential pre-industrial beginnings [96], by helping to date and document human impacts on rapidly changing environments, for example [97–99].

Establishing a high-resolution, long-term faunal record for the island of Zanzibar is critical to current conservation efforts in the heavily human-impacted Zanzibar archipelago [100–102; 35]. Resource managers and community-based conservation projects often draw on historical ecological data to model “ideal” biotic compositions (see discussion in [15]), and deep archaeofaunal and archaeobotanical records are key to such efforts. The work presented here lays the foundation for a more robust reconstruction of transformations to Zanzibar’s faunal and floral biota, and the relative contributions of human agency versus other factors in bringing them about. However, Kuumbi Cave is only a small entrée into the potentially widespread and complex processes of prehistoric and historic defaunation across Zanzibar, and a number of issues remain unresolved. Amongst these are the possible impacts of middle Holocene climate change, which have not been considered here, given the absence of paleoecological and archaeological data for this period on Zanzibar. This era was, however, more broadly characterized by pervasive and rapid pulses of change across the region, such as the strong aridity shift at 4 ka noted above. This aridity likely affected Zanzibar’s ecosystem composition and distribution, and may have impacted plant and animal communities. Future research should strive to determine the nature and chronology of both human and natural impacts to Zanzibar’s flora and fauna, as well as explore in greater depth the intersection of island biogeography and human occupation during the nine millennia not represented in the Kuumbi Cave stratigraphy. Such an interdisciplinary approach should draw on currently lacking high-resolution microfaunal, paleobotanical, sedimentary, and isotopic records.

## Methods

### Model of relative sea level

The model used here considers only the sea level change associated with the growth and decay of ice sheets and includes the effects of changing ocean volume and the associated changes in gravity, the migration of shorelines, and changes in seafloor bathymetry. This model has been tested and calibrated against a global dataset from areas that are understood to be free of tectonic or other geological uplift or subsidence processes using the criteria of the last interglacial being near present sea levels, absence of seismicity, and avoidance of sites near active plate margins [75]. The confidence envelope for the model includes the contributions from the predicted spatial variability between the sites (the three coring sites and Kuumbi Cave), from the uncertainty in earth-model parameters (elastic lithosphere thickness from 50 to 80 km, upper mantle viscosity from 1.10^20^ to 2.10^20^ Pa s, and lower mantle viscosities from 1.10^22^ to 20.10^22^ Pa s, as explained in [75]) and ice-model uncertainties. Late Quaternary (perhaps to c. 250 ka) land uplift, at an estimated rate of ca. 0.10–0.20 mm/yr, has been inferred for the area around Kuumbi Cave [46] and has been adopted as the range of error due to vertical tectonic movements, and is added to the observation errors linked to instrumental uncertainties and compaction [42].

### Mangrove sedimentary records

Holocene mangrove dynamics were reconstructed using pollen analysis [40–42; 61]. These studies also revealed through charcoal analysis how past fire, climate and anthropogenic activities impacted mangrove ecosystems. Holocene RSL reconstruction was produced through a combination of an indicative meaning of lower and upper limit of mangroves and an assessment of modern pollen-vegetation and vegetation to altitudinal relationships [42].

### Faunal recovery and zooarchaeological analysis

Fieldwork at Kuumbi Cave was carried out under a permit issued by the Office of Chief Government Statistician, Zanzibar Research Committee, and an excavation license issued by the Department of Museums and Antiquities (DAMA). Field methods are described in detail elsewhere [44]. All deposits from Trench 10 were dry-sieved on site using 3 mm mesh, except for sub-samples of between 7–60L per context; these were bagged separately and processed by flotation to recover archaeobotanical remains and subsequently wet-sieved through 1mm mesh. The wet- and dry-sieved faunal samples (excluding land and marine molluscs and fish) are included in the present analysis, for a total of 17.6 kg of tetrapod faunal remains. Faunal remains are in a temporary storage facility at the British Institute in Eastern Africa, Nairobi, where they are marked with Sealinks Project catalog numbers in the range 3750–14011. This assemblage will ultimately be accessioned and accessible at the Museum of History and Culture of Zanzibar and the Swahili Coast in Stone Town, Zanzibar.

All “maximally identifiable” bones (teeth, epiphyses, other highly diagnostic specimens) were recorded, and in 11 out of 23 contexts, all “minimally identifiable” bones (limb shafts and axial fragments >2 cm) were also recorded. Variables recorded included taxon, skeletal element, portion, side, and where possible, estimated age; in the subset of 11 contexts, a range of taphonomic variables were also recorded (S2 Appendix). Taxonomic identifications (S1 Table) were made using the reference collections of the National Museums of Kenya, Nairobi, as well as, to a lesser extent, standard guides such as [103]. Mammal size classes were adapted from [104], where Size 1 includes suni and duikers; Size 2 includes domestic caprines, bushpig, bushbuck and reedbuck; Size 3 includes domestic cattle, waterbuck and zebra; and Size 4 includes buffalo. Estimates of the Minimum Number of Individuals (MNI) took into account laterality, size, and where relevant, age estimates. It should be stressed that limb shafts were not studied in all contexts and therefore could not be used to calculate the Minimum Number of Elements (MNE) and resulting MNI values, as is standard practice in contexts where density-mediated attrition has taken place [105]. Therefore, MNI estimates may be too low in some cases.

## Supporting Information

S1 Appendix Radiometric dating for Trench 10, Kuumbi Cave. (DOCX)

S2 Appendix Taphonomic variables recorded in the Kuumbi Cave Trench 10 assemblage. (DOCX)

S1 Fig Diversity indices at Kuumbi Cave. Diversity indices (Shannon’s E, Simpson’s Dominance; left axis), and richness (NTAXA; right axis), calculated by phase using both NISP and MNI.
(TIF)

S1 Table Taxonomic representation at Kuumbi Cave Trench 10. (XLSX)
