# Supplementary material for: Continental Island Formation and the Archaeology of Defaunation on Zanzibar, Eastern Africa
Source: PLoS One. 2016 Feb 22;11(2):e0149565. doi: 10.1371/journal.pone.0149565 (PMC4763145; doi:10.1371/journal.pone.0149565)
Supplement: S1 Appendix — (DOCX) [file pone.0149565.s001.docx]

**S1 Appendix. Radiometric dating for Trench 10, Kuumbi Cave.**

Twenty samples from Trench 10 at Kuumbi Cave were dated via accelerator mass spectrometry (AMS) radiocarbon dating and Optically Stimulated Luminescence (OSL) at the Oxford Radiocarbon Accelerator Unit (lab numbers beginning with X or OxA) or the University of Waikato Radiocarbon Facility (lab numbers beginning with Wk). Details of the laboratory methods and the mixed calibration curve employed (70% SHCal13, 30% IntCal13) are outlined in Shipton et al (43). As discussed in that paper, archaeological, geoarchaeological, and chronometric information suggested that most charcoal dates reflected sedimentary deposition events rather than cultural events; therefore, Kuumbi Cave’s chronology is based primarily on dates (in bold font) on human bone, ceramic and shell, which can be clearly linked to human occupation.

Ch, charcoal; Pot, pottery; MSh, marine shell; Sh, terrestrial shell; Bo, human bone. Uncal/cal bp/BP, uncalibrated/calibrated years before present.

| **Phase** | **Context** | **Material** | **Method** | **Uncal bp** | **Cal BP** | **Lab No.** |
| --- | --- | --- | --- | --- | --- | --- |
| 1b | 1003 | Ch | AMS | 5332 ± 20 | 6185-5995 | Wk-40631 |
| **1b** | **1003** | **Pot** | **OSL** | **590 ± 50** | **640-540** | **X6696P** |
| 1b | 1004 | Ch | AMS | 4467 ± 21 | 5280-5880 | Wk-40635 |
| 1b | 1004C | Ch | AMS | 1622 ± 21 | 1535-1415 | Wk-40964 |
| 1b | 1007 | Ch | AMS | 4459 ± 20 | 5275-4875 | Wk-40636 |
| **1b** | **1007** | **Pot** | **OSL** | **1360 ± 125** | **1485-1235** | **X6697P** |
| **1b** | **1007** | **Pot** | **OSL** | **685 ± 85** | **770-600** | **X6698P** |
| 1b | 1008 | Ch | AMS | 3100 ± 22 | 3360-3210 | Wk-40963 |
| 1b | 1011 | Ch | AMS | 4887 ± 20 | 5645-5490 | Wk-40634 |
| **1b** | **1011** | **Pot** | **OSL** | **960 ± 80** | **1040-880** | **X6699P** |
| **1b** | **1011** | **Bo** | **AMS** | **1479 ± 23** | **1370-1300** | **OxA-31427** |
| 2 | 1015 | Ch | AMS | 5082 ± 23 | 5900–5730 | Wk-40962 |
| **2** | **1015** | **MSh** | **AMS** | **10549 ± 35** | **12620-12410** | **Wk-42253** |
| 2 | 1016 | Ch | AMS | 1899 ± 20 | 1875-1740 | Wk-40961 |
| **2** | **1016** | **MSh** | **AMS** | **10069 ± 32** | **11750-11340** | **Wk-42254** |
| **2** | **1017** | **MSh** | **AMS** | **11082 ± 37** | **13040-12790** | **Wk-42255** |
| **2** | **1017** | **MSh** | **AMS** | **10582 ± 35** | **12640-12420** | **Wk-42256** |
| **3** | **1019** | **Sh** | **AMS** | **15460 ± 65** | **18830-18555** | **OxA-30467** |
| 3 | 1019 | Ch | AMS | 14221 ± 62 | 17485-17080 | Wk-40632 |
| 4 | 1025 | Ch | AMS | 16656 ± 56 | 20240-19880 | Wk-40633 |
