# Supplementary material for: Continental Island Formation and the Archaeology of Defaunation on Zanzibar, Eastern Africa
Source: PLoS One. 2016 Feb 22;11(2):e0149565. doi: 10.1371/journal.pone.0149565 (PMC4763145; doi:10.1371/journal.pone.0149565)
Supplement: S2 Appendix — (DOCX) [file pone.0149565.s002.docx]

**S2 Appendix.** Taphonomic variables recorded in the Kuumbi Cave Trench 10 assemblage.

**Analytical methods:**

Selected contexts were subjected to a detailed taphonomic analysis. All specimens identifiable to, at minimum, skeletal element, were recorded, including all limb shafts >2 cm in length. Cortical visibility was rated as “good” (complete surface visible), “moderate” (>50% of surface visible) or “poor” (<50% visible). Each specimen was then examined with a 20x hand lens under strong oblique light to search for marks. Marks sought include cut marks, carnivore and rodent tooth marks, percussion marks, and sedimentary abrasion. Limb shafts were examined for breakage patterns, with fracture planes classified as green (fresh) or diagenetic (dry). Notches on breakage planes were examined to determine if these were created via hammerstone percussion (dynamic loading) or the action of carnivores (static loading). Burning was recorded for the full Trench 10 assemblage, not only the contexts noted above.

**Table S2.1.** Bone surface modifications recorded in the selected contexts.

|  |  | **CM^1^** | | **CM + CM?^2^** | | **TM** | | **PM** | | **RM** | | **Abrasion** | |
| --- | --- | --- | --- | --- | --- | --- | --- | --- | --- | --- | --- | --- | --- |
| **Context** | **NISP** | **NISP** | **%** | **NISP** | **%** | **NISP** | **%** | **NISP** | **%** | **NISP** | **%** | **NISP** | **%** |
| 1004 | 570 | 4 | 0.7 | 4 | 0.7 |  | 0.0 |  | 0.0 | 3 | 0.5 | 12 | 2.1 |
| 1007 | 841 | 3 | 0.4 | 3 | 0.4 | 2 | 0.2 |  | 0.0 | 2 | 0.2 | 10 | 1.2 |
| 1011 | 352 | 2 | 0.6 | 4 | 1.1 | 1 | 0.3 | 1 | 0.3 |  | 0.0 | 2 | 0.6 |
| 1012 | 24 |  | 0.0 |  | 0.0 |  | 0.0 |  | 0.0 |  | 0.0 |  | 0.0 |
| 1013 | 63 | 1 | 1.6 | 2 | 3.2 |  | 0.0 |  | 0.0 |  | 0.0 | 1 | 1.6 |
| 1016 | 140 | 2 | 1.4 | 2 | 1.4 |  | 0.0 |  | 0.0 |  | 0.0 | 1 | 0.7 |
| 1017 | 802 | 11 | 1.4 | 15 | 1.9 |  | 0.0 |  | 0.0 | 3 | 0.4 | 6 | 0.7 |
| 1019 | 1302 | 23 | 1.8 | 40 | 3.1 | 3 | 0.2 |  | 0.0 | 2 | 0.2 | 10 | 0.8 |
| 1021 | 11 |  | 0.0 |  | 0.0 |  | 0.0 |  | 0.0 |  | 0.0 |  | 0.0 |
| 1024 | 981 | 2 | 0.2 | 5 | 0.5 |  | 0.0 |  | 0.0 | 3 | 0.3 | 10 | 1.0 |
| 1026 | 32 |  | 0.0 |  | 0.0 |  | 0.0 |  | 0.0 |  | 0.0 |  | 0.0 |
| TOTAL | 5118 | 48 | 0.9 | 75 | 1.5 | 6 | 0.1 | 1 | 0.2% | 13 | 0.3 | 52 | 1.0 |

CM, Cut mark; TM, (likely carnivore) tooth pit; PM, percussion mark; RM, rodent tooth mark

^1^ Definitive CM

^2^ Includes definitive and ambiguous CM

**Table S2.2.** Cut marks on the subsample of specimens graded as having “good” or “moderate” cortical visibility, and excluding specimens graded as having “poor” visibility.

|  |  | **CM^1^** | | **CM + CM?^2^** | |
| --- | --- | --- | --- | --- | --- |
| **Context** | **NISP** | **NISP** | **%** | **NISP** | **%** |
| 1004 | 119 | 2 | 2 | 3 | 3 |
| 1007 | 173 | 3 | 2 | 3 | 2 |
| 1011 | 52 | 1 | 2 | 3 | 6 |
| 1013 | 16 | 1 | 6 | 2 | 13 |
| 1016 | 66 | 1 | 2 | 1 | 2 |
| 1017 | 292 | 8 | 3 | 12 | 4 |
| 1019 | 664 | 22 | 3 | 39 | 6 |
| 1024 | 370 | 1 | 0.3 | 3 | 1 |
| TOTAL | 1752 | 39 | 2.2 | 66 | 4 |

^1^ Definitive CM; ^2^ Includes definitive and ambiguous CM

**Table S2.3.** Cut mark frequencies by carcass size.

|  | **Carcass Sizes 0.5-1/2^1^** | | | **Carcass Sizes 2-4^2^** | | |
| --- | --- | --- | --- | --- | --- | --- |
| **Context** | **NISP** | **CM** | % | **NISP** | **CM** |  |
| 1004 | 537 | 4 | 0.7 | - |  |  |
| 1007 | 780 | 3 | 0.4 | - |  |  |
| 1011 | 332 | 1 | 0.3 | 11 | 1 | 9 |
| 1013 | 61 | 1 | 2 | - |  |  |
| 1016 | 127 | 2 | 2 | - |  |  |
| 1017 | 644 | 7 | 1 | 109 | 4 | 4 |
| 1019 | 1006 | 12 | 1 | 278 | 11 | 4 |
| 1024 | 819 | 2 | 0.2 | - |  |  |
| TOTAL | 4306 | 32 | 1 | 398 | 16 | 4 |

^1^ Examples of Sizes 0.5, 1, and 1-2: hyrax, giant rat, monkey, suni, duiker

^2^ Examples of Sizes 2, 3, and 4: bushpig, bushbuck, reedbuck, waterbuck, zebra, buffalo

**Table S2.4.** Notches observed in the taphonomic subsample, by agent of breakage.

|  |  | **Carnivore** | | **Percussion** | | **Unknown** | | **Total** | |
| --- | --- | --- | --- | --- | --- | --- | --- | --- | --- |
| **Context** | **NISP^1^** | **NISP** | **%** | **NISP** | **%** | **NISP** | **%** | **NISP** | **%** |
| 1004 | 217 | 6 | 3 |  |  | 3 | 1 | 9 | 4 |
| 1007 | 322 | 2 | 1 |  |  | 5 | 2 | 7 | 2 |
| 1011 | 84 |  |  | 1 | 1 | 1 | 1 | 2 | 2 |
| 1012 | 8 |  |  |  |  |  |  | 0 | 0 |
| 1013 | 28 |  |  |  |  |  |  | 0 | 0 |
| 1016 | 63 |  |  |  |  |  |  | 0 | 0 |
| 1017 | 354 |  |  |  |  | 3 | 1 | 3 | 1 |
| 1019 | 471 | 1 | 0 |  |  | 1 | 0.2 | 2 | 0 |
| 1021 | 7 |  |  |  |  |  |  | 0 | 0 |
| 1024 | 374 |  |  |  |  | 3 | 1 | 3 | 1 |
| 1026 | 22 |  |  |  |  |  |  | 0 | 0 |
| TOTAL | 1913 | 9 | 0.5 | 1 | 0.1 | 16 | 1 | 26 | 1 |

^1^ NISP includes shafts of long limb bones (humerus, femur, radio-ulna, tibia, metapodials)

**Table S2.5.** Breakage plane types.

|  |  | **Diagenetic** | | **Green** | | **Modern** | |
| --- | --- | --- | --- | --- | --- | --- | --- |
| **Context** | **NISP^1^** | **NISP** | **%** | **NISP** | **%** | **NISP** | **%** |
| 1004 | 135 | 40 | 30 | 86 | 64 | 28 | 21 |
| 1007 | 168 | 22 | 13 | 115 | 68 | 54 | 32 |
| 1011 | 38 | 7 | 18 | 32 | 84 | 1 | 3 |
| 1012 | 7 | 1 | 14 | 2 | 29 | 5 | 71 |
| 1013 | 14 | 2 | 14 | 7 | 50 | 5 | 36 |
| 1016 | 16 | 2 | 13 | 14 | 88 | 0 | 0 |
| 1017 | 165 | 26 | 16 | 129 | 78 | 27 | 16 |
| 1019 | 330 | 16 | 5 | 214 | 65 | 115 | 35 |
| 1021 | 4 | 3 | 75 | 1 | 25 | 0 | 0 |
| 1024 | 244 | 13 | 5 | 190 | 78 | 79 | 32 |
| 1026 | 15 | 3 | 20 | 13 | 87 | 1 | 7 |
| TOTAL | 1136 | 135 | 12 | 803 | 71 | 315 | 28 |

^1^ NISP includes shafts of long limb bones (humerus, femur, radio-ulna, tibia, metapodials) for which breakage plane types could be determined and were recorded. Some specimens exhibit more than one breakage type, therefore percentages do not add up to 100%.

**Table S2.6.** Burned bone in the full Trench 10 assemblage (all contexts).

| **Context** | **NISP^1^** | **NISP Burned** | **%** |
| --- | --- | --- | --- |
| 1001 | 16 | 4 | 25 |
| 1002 | 134 | 4 | 3 |
| 1003 | 313 | 29 | 9 |
| 1004 | 570 | 101 | 18 |
| 1005 | 37 | 1 | 3 |
| 1007 | 841 | 67 | 8 |
| 1008 | 34 | 4 | 12 |
| 1010 | 30 | 12 | 40 |
| 1011 | 352 | 57 | 16 |
| 1012 | 24 | 2 | 8 |
| 1013 | 63 | 8 | 13 |
| 1015 | 221 | 47 | 21 |
| 1016 | 140 | 14 | 10 |
| 1017 | 802 | 219 | 27 |
| 1018 | 325 | 26 | 8 |
| 1019 | 1302 | 112 | 9 |
| 1020 | 65 | 23 | 35 |
| 1021 | 11 | 7 | 64 |
| 1022 | 66 | 14 | 21 |
| 1023 | 21 | 2 | 10 |
| 1024 | 981 | 122 | 12 |
| 1025 | 114 | 4 | 4 |
| 1026 | 32 | 1 | 3 |
| Total | 6494 | 880 | 14 |

^1^ NISP excludes teeth
