# Supplementary figures and images for: Continental Island Formation and the Archaeology of Defaunation on Zanzibar, Eastern Africa
Source: PLoS One. 2016 Feb 22;11(2):e0149565. doi: 10.1371/journal.pone.0149565 (PMC4763145; doi:10.1371/journal.pone.0149565)

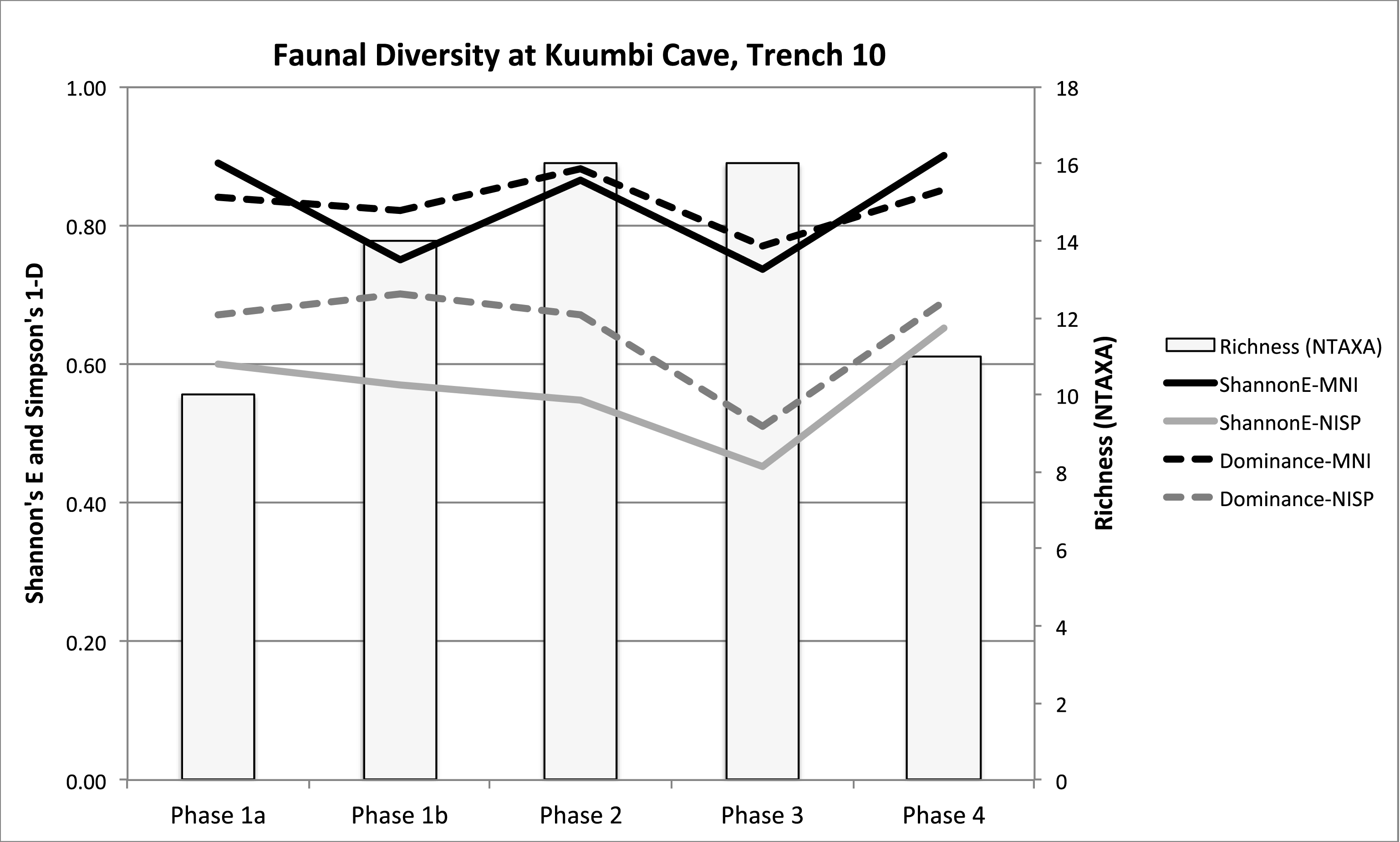

Supplement: S1 Fig — Diversity indices (Shannon’s E, Simpson’s Dominance; left axis), and richness (NTAXA; right axis), calculated by phase using both NISP and MNI. (TIF) [file pone.0149565.s003.tif]
